# Effects of exercise interventions on leptin and ghrelin levels in middle-aged and older adults with overweight or obesity: a systematic review and meta-analysis

**DOI:** 10.7717/peerj.21870

**Published:** 2026-11-03

**Authors:** Zhuangzhuang Guo, Wei Guo, Yimeng Gu, Lizhen Ning, Yongheng Zhao

**Affiliations:** 1 School of Wushu, Henan University, Kaifeng, China; 2 Graduate School, Kyungil University, Gyeongsan-si, Republic of South Korea

**Keywords:** Exercise, Leptin, Ghrelin, Overweight or obesity, Middle-aged and older adults, Meta-analysis

## Abstract

**Background:**

Overweight and obesity are common among middle-aged and older adults, and obesity is a chronic, multifactorial disease that, particularly when accompanied by central adiposity or metabolic abnormalities, is associated with increased cardiometabolic, cancer-related, musculoskeletal, and functional risks. Leptin and ghrelin are key hormones involved in energy balance regulation and may be dysregulated in obesity, but evidence regarding the effects of exercise interventions on leptin and ghrelin levels in middle-aged and older adults with overweight or obesity remains inconsistent. This review aimed to evaluate the effects of exercise interventions on leptin and ghrelin levels in this population.

**Methods:**

PubMed, Web of Science, Embase, the Cochrane Library, and CNKI were searched from database inception to 5 July 2026. Eligible studies were randomized controlled trials that enrolled middle-aged or older adults with overweight or obesity and reported leptin or ghrelin levels before and after an exercise intervention. Standardized mean differences (SMDs) and 95% confidence intervals (CIs) were synthesized using Review Manager 5.4 and Stata 15. Statistical heterogeneity was assessed using *I*^2^; a fixed-effect model was used when *I*^2^ < 50%, and a random-effects model when *I*^2^ ≥ 50%. Subgroup analyses, sensitivity analyses, and publication-bias assessments were conducted.

**Results:**

Twenty-one randomized controlled trials were included. Exercise interventions were associated with significantly lower leptin levels than control conditions based on 18 comparisons (SMD = −0.32, 95% CI [−0.47 to −0.18], *P* < 0.0001), with low between-study heterogeneity (*I^2^* = 22%). Exercise interventions were also associated with significantly higher ghrelin levels based on nine comparisons (SMD = 0.45, 95% CI [0.11–0.79], *P* = 0.01), although heterogeneity was substantial (*I*^2^ = 74%). Subgroup analyses suggested that exercise-related characteristics may partly contribute to variability in leptin and ghrelin responses, but these findings were exploratory. Leave-one-out sensitivity analyses supported the stability of the main estimates. For leptin, Egger’s test suggested possible funnel-plot asymmetry, but trim-and-fill analysis did not impute any missing comparison and the pooled estimate remained unchanged.

**Conclusion:**

Exercise interventions were associated with reduced leptin levels and increased ghrelin levels in middle-aged and older adults with overweight or obesity. The leptin finding appeared relatively consistent, whereas the ghrelin finding should be interpreted cautiously because of fewer comparisons and substantial heterogeneity. Subgroup findings remain exploratory and should not be used to define an optimal exercise prescription. Further randomized controlled trials with more standardized exercise protocols, clearer dietary control, and consistent hormone measurement are needed to clarify the clinical relevance of these changes.

## Introduction

Overweight and obesity are common among middle-aged and older adults, but their clinical implications vary according to fat distribution, metabolic status, and comorbidities (GBD 2021 Adult BMI Collaborators, 2025). In this population, obesity, particularly central obesity or obesity accompanied by metabolic disorders, is more consistently associated with higher risks of cardiometabolic diseases, certain cancers, musculoskeletal disorders, functional decline, and reduced healthy life expectancy (Blüher, 2025; Smith, Mittendorfer & Klein, 2019). By contrast, overweight alone may have a more heterogeneous risk profile, especially when central adiposity or metabolic abnormalities are absent. These distinctions are important because middle-aged and older adults often experience age-related increases in visceral adiposity, reductions in skeletal muscle mass and resting energy expenditure, and a higher burden of insulin resistance and multimorbidity (Prado et al., 2024). Therefore, understanding modifiable factors related to obesity-associated metabolic regulation in this age group is relevant for weight management and healthy aging.

Obesity is also accompanied by dysregulation of endocrine and neuroendocrine pathways involved in appetite and energy balance. Appetite regulation depends on coordinated signals from adipose tissue, the gastrointestinal tract, the pancreas, and the central nervous system, rather than on a single hormone (Skoracka et al., 2025). Among these signals, leptin and ghrelin have been widely studied because they represent different aspects of energy-balance regulation. Leptin is mainly secreted by adipose tissue and reflects fat mass and longer-term energy stores, whereas ghrelin is primarily secreted by the stomach and acts as an orexigenic signal related to hunger and food intake (Considine et al., 1996; Myers, Cowley & Münzberg, 2008; Müller et al., 2015). In middle-aged and older adults, age-related changes in body composition, metabolic health, gastrointestinal function, and neuroendocrine axes may further modify leptin and ghrelin responses. Lifestyle modification remains a cornerstone of obesity management, and structured exercise is an important component because it can increase energy expenditure, improve body composition, enhance insulin sensitivity, and support skeletal muscle and cardiometabolic health (Jakicic et al., 2024).

However, the effects of exercise interventions on leptin and ghrelin levels remain insufficiently characterized in middle-aged and older adults with overweight or obesity. Previous studies have generally reported reductions in leptin after exercise, whereas findings for ghrelin have been less consistent, with increases, decreases, or no clear changes reported across trials (Dorling et al., 2018; Jin et al., 2025). These discrepancies may be partly related to differences in exercise modality, intervention duration, weekly frequency, session duration, dietary control, participant characteristics, the handling of baseline values, and hormone measurement methods (King et al., 2012). Evidence is also limited regarding whether aerobic exercise, resistance training, or combined exercise produces different leptin and ghrelin responses in this population. Therefore, this systematic review and meta-analysis aimed to evaluate the effects of exercise interventions on leptin and ghrelin levels in middle-aged and older adults with overweight or obesity. Subgroup analyses were performed to explore whether exercise-related characteristics may contribute to variability in these responses. The findings may be relevant to clinicians, researchers, and public health professionals interested in exercise, obesity, and metabolic regulation in middle-aged and older adults.

## Research methods

### Study registration and reporting guidelines

This systematic review and meta-analysis was prospectively registered in the International Prospective Register of Systematic Reviews (PROSPERO; registration number: CRD420251233585; https://www.crd.york.ac.uk/PROSPERO/). The review was conducted and reported in accordance with the PRISMA 2020 statement (Page et al., 2021).

### Inclusion and exclusion criteria

Eligibility criteria were defined according to the PICOS framework (Higgins et al., 2024). Participants were middle-aged and older adults with overweight or obesity, defined according to World Health Organization or national diagnostic criteria (World Health Organization, 2000). Studies were eligible if participants were aged ≥45 years or if the baseline mean or median age was ≥45 years. Studies involving participants with obesity-related metabolic conditions, such as type 2 diabetes or metabolic syndrome, were considered eligible if participants met the overweight or obesity criteria and the other eligibility criteria. Eligible interventions were planned exercise training programs, including aerobic exercise, resistance training, or combined exercise, with an intervention duration of at least 4 weeks to exclude acute or very short-term exercise responses. Exercise interventions could be delivered alone or combined with non-exercise components, such as dietary control, provided that these components were comparable between the intervention and control groups. Eligible control conditions included no exercise intervention, usual lifestyle, usual care, health education, or low-intensity activities such as stretching, provided that they did not constitute planned exercise training. The outcomes of interest were pre- and post-intervention levels of leptin and/or ghrelin. Only randomized controlled trials were included.

Studies were excluded if they were non-randomized studies, review articles, conference abstracts, degree theses, animal studies, acute exercise studies, or studies without accessible full texts or sufficient outcome data. Studies involving special populations, such as pregnant women, postoperative patients, or individuals with eating disorders, including binge-eating disorder, bulimia nervosa, or anorexia nervosa, were also excluded.

### Literature search strategy

A comprehensive systematic search was conducted in PubMed, Web of Science, Embase, the Cochrane Library, and the China National Knowledge Infrastructure (CNKI) from database inception to 5 July 2026. No language restrictions were applied during the database searches or study-selection process. Database-specific controlled vocabulary terms and free-text keywords were combined using Boolean operators. The search strategy included terms related to exercise training, overweight or obesity, middle-aged and older adults, leptin, and ghrelin. The search strategy was adapted for each database, and the complete search strategies are provided in the Supplemental Material.

### Study selection

Two independent reviewers (ZG and YZ) screened the titles and abstracts of retrieved records according to the predefined eligibility criteria. Potentially eligible articles were then assessed in full text. Disagreements were resolved through discussion, and if consensus could not be reached, a third reviewer (WG) was consulted.

### Data extraction

Data were independently extracted by two reviewers using a standardized data extraction form created in Microsoft Excel. Extracted information included first author, publication year, country or region, participant characteristics, sample size, sex, age, intervention and control conditions, exercise type, intervention duration, weekly training frequency, session duration, and outcome data for leptin and/or ghrelin levels before and after the intervention. When available, change-from-baseline data were extracted. If outcome data were incomplete or unclear, attempts were made to contact the corresponding authors for additional information. Studies were excluded if the data required for effect size calculation could not be obtained.

### Risk of bias assessment

Risk of bias in each included study was independently assessed by two researchers (ZG and YZ) using the Cochrane Risk of Bias tool. The domains assessed were random sequence generation, allocation concealment, blinding of participants and personnel, blinding of outcome assessment, incomplete outcome data, selective reporting, and other potential sources of bias. For the other bias domain, baseline comparability, intervention implementation, and study-specific issues described in the original reports were considered. Disagreements were resolved through discussion, with consultation of a third researcher (WG) when needed.

### Statistical analysis

Statistical analyses were performed using Review Manager 5.4 (RevMan 5.4; The Cochrane Collaboration; available at: https://revman.cochrane.org/) and Stata 15 (StataCorp LLC; available at: https://www.stata.com/stata15/). Standardized mean differences (SMDs) with 95% confidence intervals (CIs) were used as effect size estimates. Change-from-baseline values were used when available; for studies that did not report standard deviations of change scores, these values were estimated from baseline and post-intervention data using an assumed correlation coefficient of 0.5. Statistical heterogeneity was assessed using *I^2^*; a fixed-effect model was used when *I*^2^ < 50%, and a random-effects model when *I*^2^ ≥ 50%. Potential sources of heterogeneity were explored through subgroup analyses based on exercise-related characteristics, including exercise type, intervention duration, weekly frequency, and session duration. The subgroup cut-offs were based on common exercise-prescription categories and the distribution of the included studies, rather than on the statistical significance of the pooled results. For multi-arm trials, relevant intervention arms were combined or shared control groups were adjusted to avoid double counting. Sensitivity analyses were performed by sequentially excluding each comparison. Publication bias was assessed using funnel plots, Begg’s test, and Egger’s test when at least 10 comparisons were available; trim-and-fill analysis was used to evaluate the influence of potential publication bias.

## Results

### Literature screening results

A total of 1,979 records were identified through database searches, including PubMed (*n* = 208), Web of Science (*n* = 859), Embase (*n* = 345), the Cochrane Library (*n* = 313), and CNKI (*n* = 254). After removal of 447 duplicate records, 1,532 records were screened by title and abstract, of which 1,407 were excluded. Subsequently, 125 reports were sought for retrieval, and all full-text reports were retrieved and assessed for eligibility. Of these, 104 reports were excluded for the following reasons: non-randomized studies (*n* = 22), ineligible participants (*n* = 25), ineligible intervention or control condition (*n* = 23), no leptin or ghrelin outcome (*n* = 20), and insufficient data for effect size calculation (*n* = 14). Ultimately, 21 randomized controlled trials were included in the systematic review and meta-analysis. The study selection process is presented in Fig. 1.

**Figure 1:**
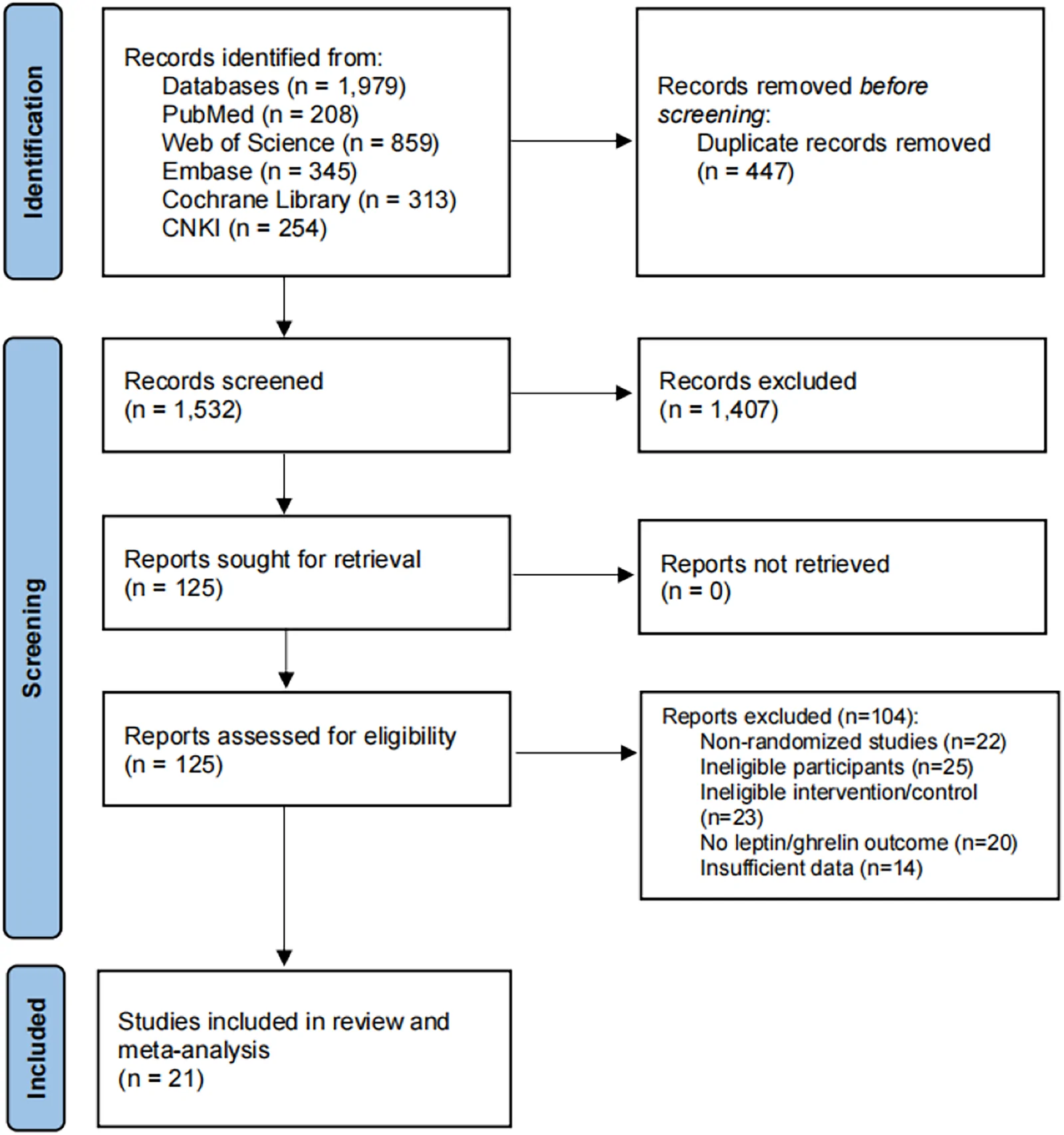
PRISMA flow diagram.

### Risk of bias assessment

The results of the risk of bias assessment are shown in Figs. 2 and 3. All 21 trials were rated as low risk for random sequence generation. Allocation concealment was rated as low risk in three trials and unclear in the remaining trials, mainly because concealment procedures were not adequately described. Because of the nature of exercise training interventions, blinding of participants and personnel was not feasible and was therefore rated as high risk across all trials. Blinding of outcome assessment was rated as low risk in most trials, although four trials provided insufficient information and were rated as unclear. For incomplete outcome data, most trials were rated as low risk, while several had unclear risk because attrition or missing outcome data were not fully reported. Selective reporting was rated as low risk in all trials. For other potential sources of bias, most trials were rated as low risk, whereas several were rated as unclear because the available reports did not provide enough detail to rule out study-specific concerns. Overall, the main concerns were the absence of participant and personnel blinding and incomplete reporting of allocation concealment.

**Figure 2:**
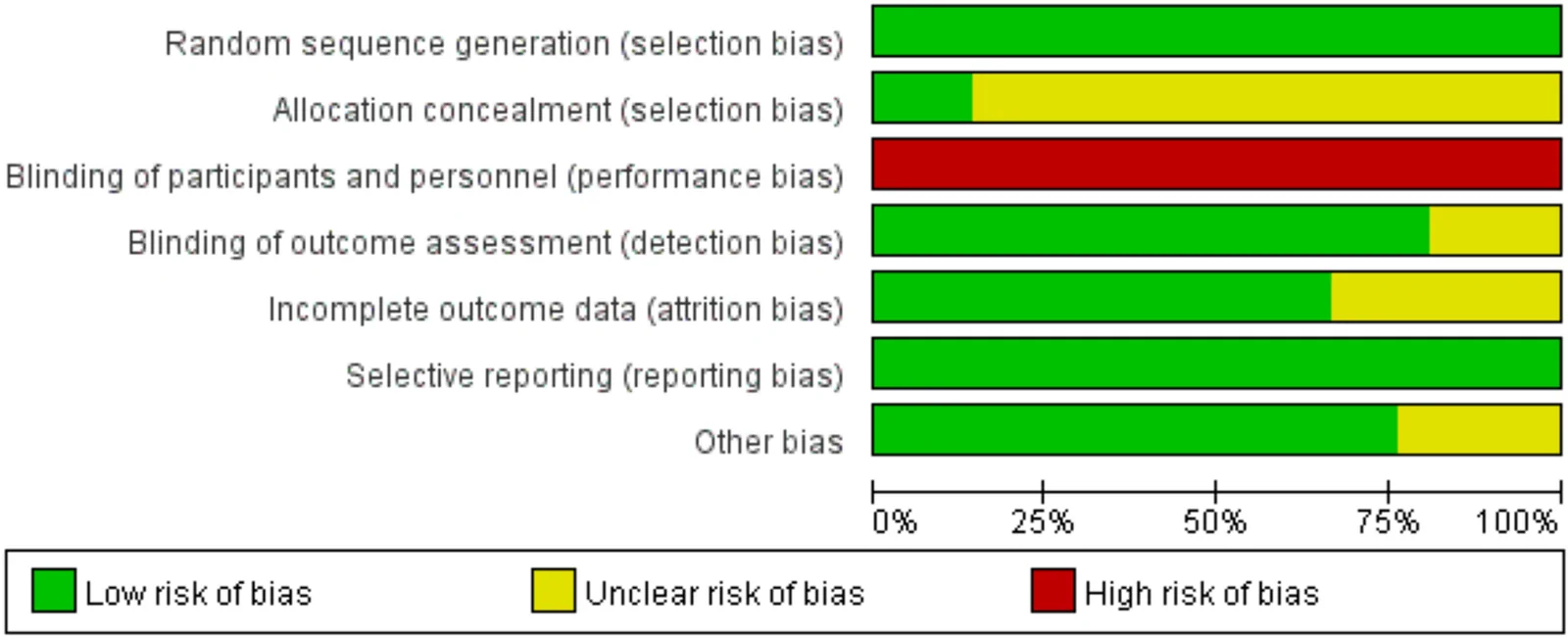
Risk of bias graph.

**Figure 3:**
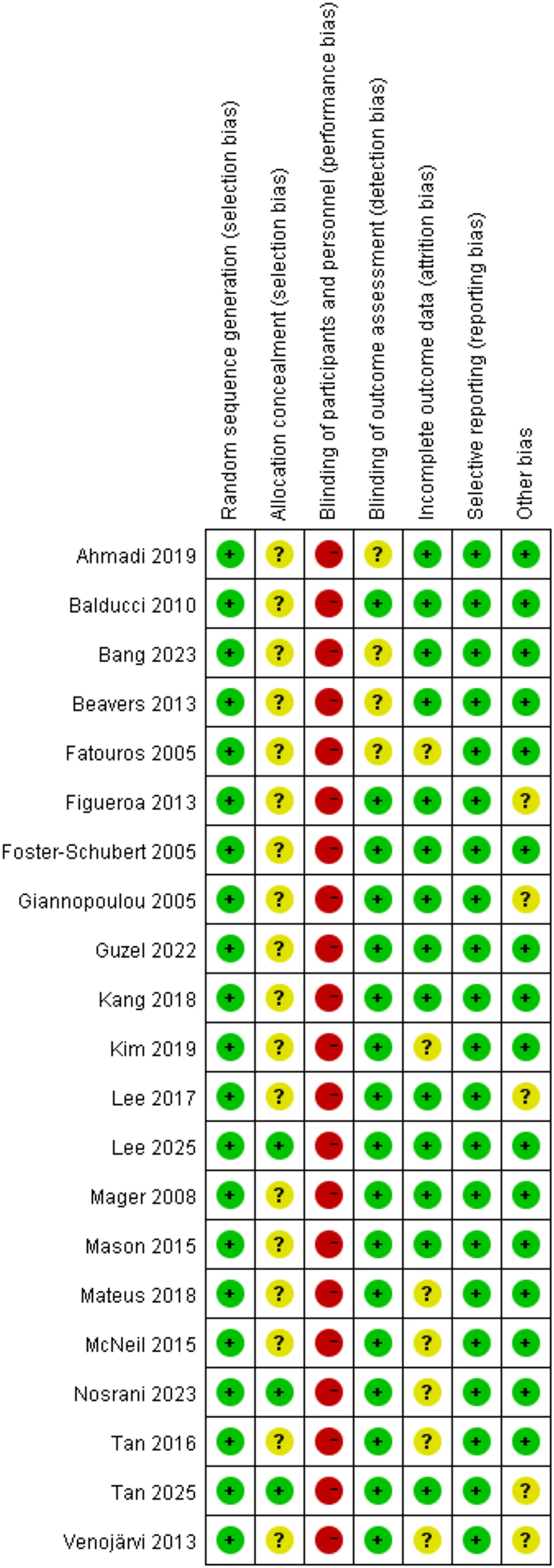
Risk of bias summary.

### Basic characteristics of included studies

The basic characteristics of the included studies are summarized in Table 1. A total of 21 randomized controlled trials published between 2005 and 2025 were included (McNeil et al., 2015; Bang, 2023; Ahmadi et al., 2019; Kang et al., 2018; Lee et al., 2017; Mateus et al., 2018; Mager et al., 2008; Foster-Schubert et al., 2005; Mason et al., 2015; Fatouros et al., 2005; Venojärvi et al., 2013; Kim et al., 2019; Tan et al., 2025; Guzel et al., 2022; Tan et al., 2016; Figueroa et al., 2013; Giannopoulou et al., 2005; Balducci et al., 2010; Beavers et al., 2013; Nosrani et al., 2023; Lee et al., 2025). The trials were conducted in different countries or regions and enrolled middle-aged or older adults with overweight or obesity. Some studies included participants with obesity-related metabolic conditions, but all met the predefined eligibility criteria. Sex composition varied across trials, with some studies recruiting women only and others including both men and women. Exercise interventions included aerobic exercise, resistance training, and combined exercise programs, with variation in intervention duration, weekly frequency, and session duration. Control conditions included usual lifestyle, usual care, health education, no exercise intervention, or low-intensity activities such as stretching. The outcome measures were leptin and/or ghrelin levels.

**Table 1:** Characteristics of included studies.

Study	Country	Sex	Age, years	*n* (IG/CG)	IG *vs*. CG	Dur. (wk)	Freq. (t/w)	Session (min)	Outcomes
McNeil et al. (2015)	Canada	Female only	Overall: 58.1 ± 4.8	28/65	IG: Resistance training + dietary restriction	24	3	NR	1
					CG: Dietary restriction				
Bang (2023)	South Korea	Female only	IG: 60.00 ± 2.97	8/8	IG: Resistance training	12	5	60	1
			CG: 60.62 ± 2.77		CG: Maintained usual lifestyle				
	South Korea	Female only	IG: 59.42 ± 2.57	7/8	IG: Resistance training	12	5	60	1
			CG: 60.62 ± 2.77		CG: Maintained usual lifestyle				
	South Korea	Female only	IG: 59.37 ± 3.20	8/8	IG: Resistance training	12	5	60	1
			CG: 60.62 ± 2.77		CG: Maintained usual lifestyle				
Ahmadi et al. (2019)	Iran	Male only	IG: 69.13 ± 3.68	15/15	IG: Aerobic exercise	8	4	50–60	2
			CG: 69.20 ± 4.31		CG: Maintained usual lifestyle				
Kang et al. (2018)	South Korea	Female only	IG: 50.1 ± 3.8	13/13	IG: Combined exercise	12	5	50	1, 2
			CG: 49.8 ± 2.9		CG: Maintained usual lifestyle				
Lee et al. (2017)	South Korea	Female only	Overall: 54.47 ± 2.50	18/18	IG: Aerobic exercise	16	3	60	1, 2
					CG: Maintained usual lifestyle				
Mateus et al. (2018)	Brazil	Male only	Overall: 49.32 ± 5.74	12/10	IG: Combined exercise	24	3	60	2
					CG: Maintained usual lifestyle				
Mager et al. (2008)	Finland	M/F = 25/22	Overall: 60 ± 7	15/18	IG: Aerobic exercise	33	4	30	2
					CG: Maintained usual lifestyle				
	Finland	M/F = 25/22	Overall: 60 ± 7	14/18	IG: Resistance training	33	4	NR	2
					CG: Maintained usual lifestyle				
Foster-Schubert et al. (2005)	USA	Female only	IG: 60.7 ± 6.7	83/85	IG: Aerobic exercise	52	5	45	2
			CG: 60.6 ± 6.8		CG: Maintained usual lifestyle				
Mason et al. (2015)	USA	Female only	Overall: 57.9 ± 5.0	105/80	IG: Aerobic exercise	52	5	45	2
					CG: Maintained usual lifestyle				
	USA	Female only	Overall: 57.9 ± 5.0	108/105	IG: Aerobic exercise + dietary restriction	52	5	45	2
					CG: Dietary restriction				
Fatouros et al. (2005)	Greece	Male only	Overall: 65–78	14/10	IG: Resistance training	24	3	NR	1
					CG: Maintained usual lifestyle				
	Greece	Male only	Overall: 65–78	12/10	IG: Resistance training	24	3	NR	1
					CG: Maintained usual lifestyle				
	Greece	Male only	Overall: 65–78	14/10	IG: Resistance training	24	3	NR	1
					CG: Maintained usual lifestyle				
Venojärvi et al. (2013)	Finland	Male only	Overall: 54–55	39/40	IG: Aerobic exercise	12	3	60	1
					CG: Maintained usual lifestyle				
	Finland	Male only	Overall: 54–55	36/40	IG: Resistance training	12	3	60	1
					CG: Maintained usual lifestyle				
Kim et al. (2019)	South Korea	Male only	Overall: 68.8 ± 0.9	10/10	IG: Combined exercise	12	3	90–120	1
					CG: Maintained usual lifestyle				
Tan et al. (2025)	UK	Female only	Overall: 56.7 ± 3.9	10/10	IG: Aerobic exercise	8	3	20	1
					CG: Maintained usual lifestyle				
Guzel et al. (2022)	Turkey	Female only	Overall: 55.0 ± 3.7	12/12	IG: Aerobic exercise	10	3	35–50	1
					CG: Maintained usual lifestyle				
Tan et al. (2016)	China	Female only	IG: 50.7 ± 5.5	15/11	IG: Aerobic exercise	10	5	60	1
			CG: 49.7 ± 7.9		CG: Maintained usual lifestyle				
Figueroa et al. (2013)	USA	Female only	Overall: 54 ± 6	14/13	IG: Resistance training + dietary restriction	12	3	30	1
					CG: Dietary restriction				
Giannopoulou et al. (2005)	USA	Female only	IG: 57.4 ± 1.7	11/11	IG: Aerobic exercise + dietary restriction	14	3–4	60	1
			CG: 58.5 ± 1.7		CG: Dietary restriction				
Balducci et al. (2010)	Italy	M/F = 37/25	Overall: 64.3 ± 8.1	20/20	IG: Aerobic exercise	52	2	60	1
					CG: Maintained usual lifestyle				
	Italy	M/F = 37/25	Overall: 60.6 ± 9.3	22/20	IG: Combined exercise	52	2	60	1
					CG: Maintained usual lifestyle				
Beavers et al. (2013)	USA	67% female	Overall: 67.0 ± 4.8	97/93	IG: Aerobic exercise	78	5	30	1
					CG: Maintained usual lifestyle				
Nosrani et al. (2023)	Portugal	M/F = 11/21	IG: 71.16 ± 5.91	19/13	IG: Combined exercise	28	2	35–50	1
			CG: 73.38 ± 5.81		CG: Maintained usual lifestyle				
Lee et al. (2025)	South Korea	Female only	IG: 56.63 ± 4.31	8/8	IG: Aerobic exercise	8	4	60	1
			CG: 56.75 ± 2.92		CG: Maintained usual lifestyle				

**Note:**

IG, intervention group; CG, control group; M/F, male/female; Dur., intervention duration; Freq., exercise frequency; wk, weeks; t/w, times per week; NR, not reported. Outcomes: 1, leptin; 2, ghrelin.

## Meta-analysis results

### Effect of exercise interventions on leptin levels

Eighteen comparisons reported leptin data and were included in the meta-analysis. Compared with control conditions, exercise interventions were associated with a significant reduction in leptin levels (SMD = −0.32, 95% CI [−0.47 to −0.18], *P* < 0.0001), with low between-study heterogeneity (*I^2^* = 22%; Fig. 4).

**Figure 4:**
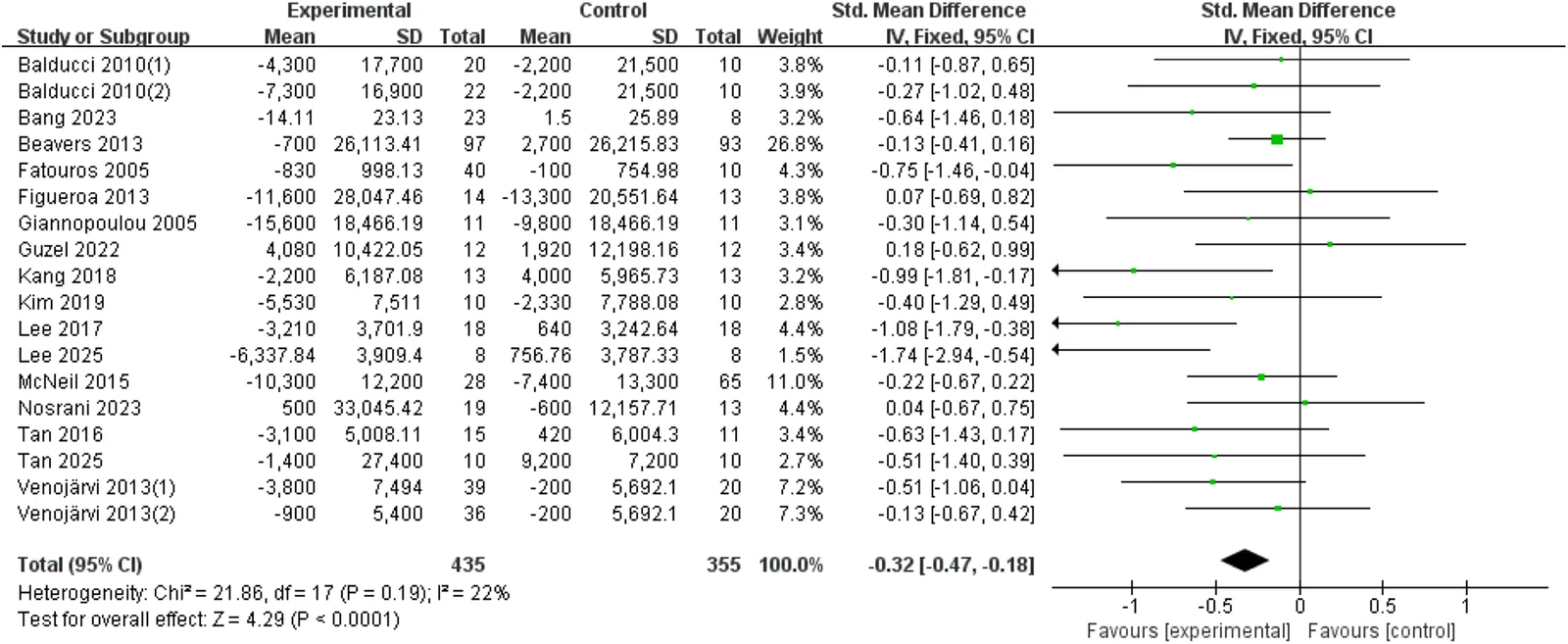
Forest plot for leptin levels.

Subgroup analyses showed broadly consistent reductions in leptin across exercise-related characteristics (Table 2). By exercise type, significant reductions were observed after aerobic exercise (SMD = −0.33, 95% CI [−0.53 to −0.14]) and resistance training (SMD = −0.28, 95% CI [−0.55 to −0.01]), whereas the estimate for combined exercise did not reach statistical significance (SMD = −0.36, 95% CI [−0.75 to 0.03]). No significant subgroup difference was observed by exercise type. Reductions in leptin were also observed across weekly frequency and intervention-duration subgroups. Among studies reporting session duration, interventions lasting ≥60 min per session showed a larger reduction than those lasting <60 min per session, although this finding should be interpreted cautiously.

**Table 2:** Subgroup analyses of leptin and ghrelin levels.

Outcome	Subgroup	*k*	SMD [95% CI]	*I* ^2^	*P*	*P subgroup*
**Leptin**	**Exercise modality**					0.94
	Aerobic exercise	9	−0.33 [−0.53 to −0.14]	45%	0.0009	
	Resistance training	5	−0.28 [−0.55 to −0.01]	0%	0.04	
	Combined exercise	4	−0.36 [−0.75 to 0.03]	15%	0.07	
	**Intervention duration**					0.30
	≤12 weeks	10	−0.42 [−0.66 to −0.18]	25%	0.0005	
	>12 weeks	8	−0.26 [−0.45 to −0.07]	20%	0.006	
	**Weekly frequency**					0.77
	≤3 sessions/week	12	−0.30 [−0.50 to −0.11]	1%	0.002	
	>3 sessions/week	6	−0.35 [−0.58 to −0.12]	53%	0.003	
	**Session duration**					0.05
	<60 min/session	6	−0.16 [−0.38 to 0.06]	15%	0.16	
	≥60 min/session	10	−0.49 [−0.72 to −0.25]	13%	<0.0001	
**Ghrelin**	**Exercise modality**					0.12
	Aerobic exercise	6	0.27 [−0.05 to 0.59]	70%	0.10	
	Resistance training	1	0.11 [−0.73 to 0.94]	–	0.81	
	Combined exercise	2	1.34 [0.34–2.34]	58%	0.009	
	**Intervention duration**					0.23
	≤12 weeks	2	1.14 [−0.22 to 2.49]	81%	0.10	
	>12 weeks	7	0.28 [−0.03 to 0.59]	67%	0.08	
	**Weekly frequency**					0.01
	≤3 sessions/week	2	1.25 [0.52–1.97]	36%	0.0007	
	>3 sessions/week	7	0.23 [−0.06 to 0.52]	61%	0.12	
	**Session duration**					0.01
	<60 min/session	6	0.25 [−0.07 to 0.57]	67%	0.12	
	≥60 min/session	2	1.25 [0.52–1.97]	36%	0.0007	

**Note:**

k, number of comparisons; SMD, standardized mean difference; CI, confidence interval; *I*^2^, heterogeneity statistic; *P* subgroup, *P*-value for subgroup differences.

### Effect of exercise interventions on ghrelin levels

Nine comparisons reported ghrelin data and were included in the meta-analysis. Compared with control conditions, exercise interventions were associated with a significant increase in ghrelin levels (SMD = 0.45, 95% CI [0.11–0.79], *P* = 0.01), with substantial between-study heterogeneity (*I^2^* = 74%; Fig. 5).

**Figure 5:**
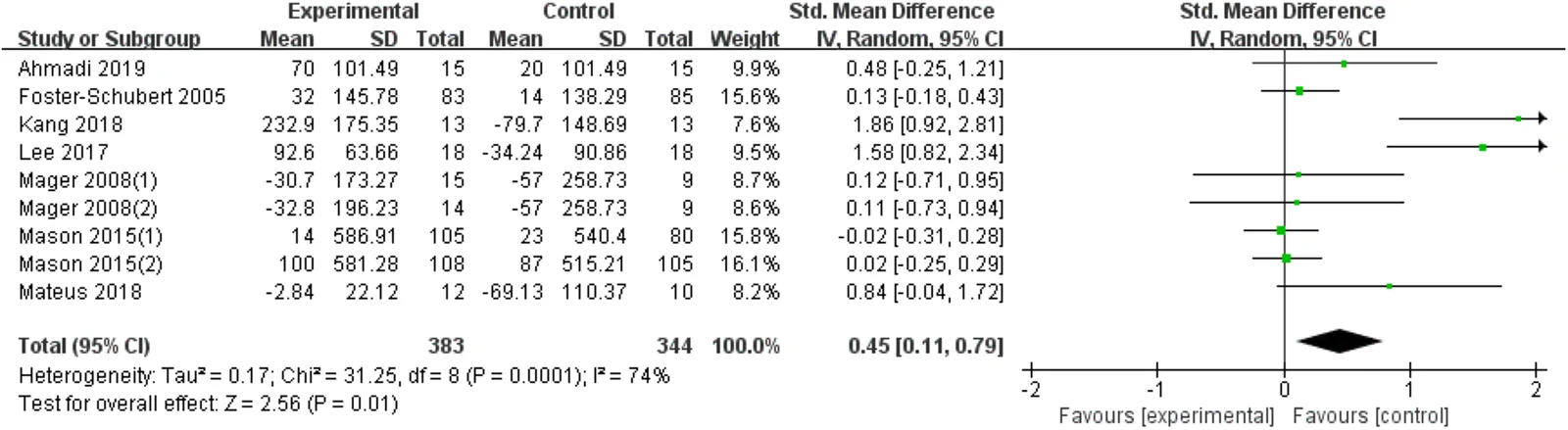
Forest plot for ghrelin levels.

Subgroup analyses suggested that ghrelin responses varied across exercise-related characteristics (Table 2). By exercise type, combined exercise was associated with a significant increase in ghrelin levels (SMD = 1.34, 95% CI [0.34–2.34]), whereas aerobic exercise and resistance training did not show statistically significant effects. However, the subgroup difference by exercise type was not significant. By weekly frequency, the ≤3 sessions/week subgroup showed a significant increase in ghrelin levels, whereas the >3 sessions/week subgroup did not, with a significant subgroup difference. Among studies reporting session duration, interventions lasting ≥60 min per session were associated with a significant increase in ghrelin levels, whereas those lasting <60 min per session were not; the subgroup difference was also significant. No significant subgroup difference was observed by intervention duration. Given the limited number of ghrelin comparisons and the substantial heterogeneity, these subgroup findings should be interpreted cautiously.

### Sensitivity analysis

Leave-one-out sensitivity analyses were performed to assess the influence of individual comparisons on the pooled estimates. For leptin, sequential exclusion of each comparison did not materially change the pooled result, and the estimate remained statistically significant. For ghrelin, the pooled estimate generally remained in the same positive direction after sequential exclusions; however, this finding should be interpreted cautiously because of the limited number of comparisons and substantial heterogeneity. The sensitivity analysis results are shown in Figs. S1 and S2.

### Publication bias

Funnel plots for leptin and ghrelin are shown in Figs. 6 and 7. For leptin, visual inspection of the funnel plot suggested possible asymmetry. Begg’s test did not provide evidence of funnel-plot asymmetry (*P* = 0.088), whereas Egger’s test suggested possible funnel-plot asymmetry (*P* = 0.043). Trim-and-fill analysis did not impute any missing comparison; therefore, no adjusted estimate different from the original pooled estimate was generated. The pooled leptin estimate remained unchanged (SMD = −0.32, 95% CI [−0.47 to −0.18]). For ghrelin, the funnel plot showed an uneven distribution of comparisons; however, because the number of available comparisons was limited, the funnel plot was interpreted descriptively only, and no firm conclusion regarding publication bias was drawn.

**Figure 6:**
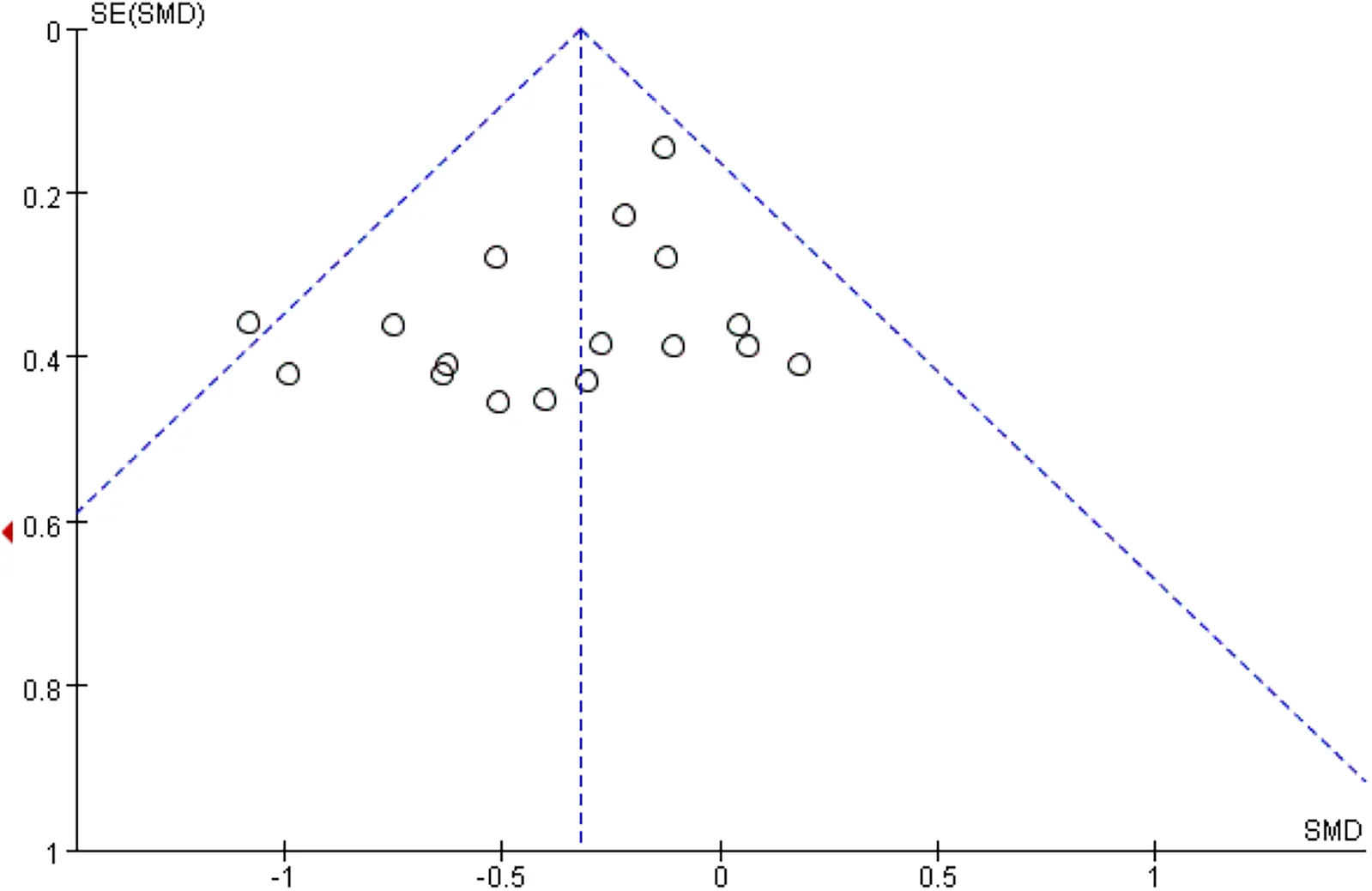
Funnel plot for leptin.

**Figure 7:**
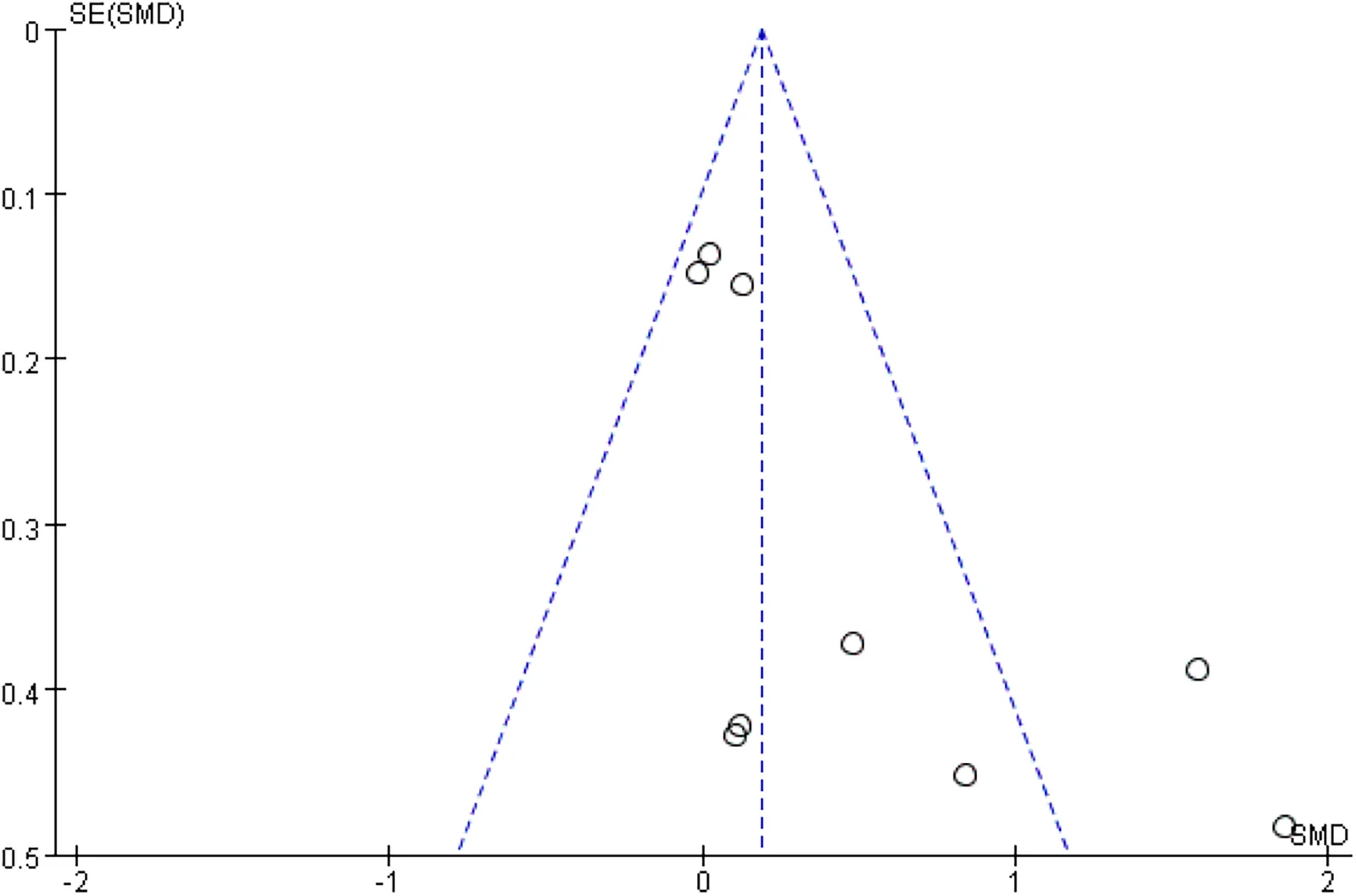
Funnel plot for ghrelin.

## Discussion

### Principal findings

This systematic review and meta-analysis included 21 randomized controlled trials evaluating the effects of exercise interventions on leptin and ghrelin levels in middle-aged and older adults with overweight or obesity. The pooled evidence indicated that exercise interventions were associated with lower leptin levels (SMD = −0.32, 95% CI [−0.47 to −0.18]; *I^2^* = 22%) and higher ghrelin levels (SMD = 0.45, 95% CI [0.11–0.79]; *I^2^* = 74%); however, the higher heterogeneity for ghrelin indicates that this finding should be interpreted more cautiously than the leptin finding. Subgroup analyses suggested that exercise-related characteristics may partly contribute to variability in hormonal responses, although these findings should be considered exploratory because several subgroup estimates were based on a limited number of comparisons. Taken together, these findings suggest that exercise is associated with directionally distinct leptin and ghrelin responses, although the inconsistency in the ghrelin evidence means that these findings should be interpreted cautiously and regarded as hypothesis-generating.

### Interpretation of leptin and ghrelin findings

In middle-aged and older adults with overweight or obesity, exercise-related reductions in leptin should be interpreted in relation to the age-associated metabolic phenotype rather than as an isolated endocrine change. In this population, excess adiposity often overlaps with visceral fat accumulation, declining skeletal muscle mass and resting energy expenditure, insulin resistance, chronic low-grade inflammation, and, in many women, postmenopausal changes in fat distribution and adipokine signaling (Villareal et al., 2005; Hotamisligil, 2006). Because leptin is primarily secreted by adipocytes and reflects longer-term adiposity and energy stores, lower leptin after exercise may indicate reduced adipose-tissue burden, altered energy availability, or a more favorable metabolic milieu. Exercise may contribute to these changes through reductions in fat mass, improvements in insulin sensitivity, attenuation of inflammatory signaling, and enhanced skeletal muscle metabolic activity (Pedersen & Febbraio, 2012; Fedewa et al., 2018). However, this interpretation must remain cautious. Obesity is commonly characterized by hyperleptinemia together with reduced leptin responsiveness, and most trials included in this review measured only leptin levels rather than leptin receptor activity, central leptin signaling, hypothalamic responsiveness, or leptin-mediated appetite behavior (Izquierdo et al., 2019). Therefore, the observed decline in leptin should not be regarded as direct evidence that exercise restored leptin sensitivity or central appetite control.

The increase in ghrelin may be understood as a complementary short-term orexigenic signal within the same energy-balance framework. Ghrelin is mainly secreted by the stomach and rises in states of energy deprivation, acting as a signal of energy need and hunger drive (Cummings et al., 2001). In the context of exercise, higher ghrelin may therefore reflect a compensatory response to increased energy expenditure, reduced energy availability, or loss of body mass (Hopkins, King & Blundell, 2010). This interpretation is particularly relevant for middle-aged and older adults because aging may alter the coupling between peripheral appetite hormones and behavioral food intake. For example, age-related appetite decline, changes in gastrointestinal function, altered body composition, and metabolic comorbidities may weaken or modify the translation of peripheral ghrelin signals into actual eating behavior (Wysokiński et al., 2015). Thus, an increase in ghrelin does not necessarily imply a uniform increase in food intake, but it may indicate a compensatory energy-balance signal. The interpretation of ghrelin is further complicated by methodological differences across trials. Differences in exercise modality, intervention duration, energy intake, fasting status, blood-sampling time, changes in body composition, and ghrelin measurement methods may all contribute to the inconsistency of ghrelin findings. Some of these intervention-level factors were further explored in the subgroup analyses, whereas other sources of variability, such as hormone assay type, sampling conditions, and energy intake, could not be quantitatively examined because they were inconsistently reported across trials.

Taken together, the opposite directions of leptin and ghrelin changes are compatible with a reciprocal energy-balance response after exercise. Leptin represents a longer-term adiposity-related signal, whereas ghrelin provides a shorter-term orexigenic signal; a reduction in leptin alongside an increase in ghrelin may therefore reflect the body’s attempt to defend energy stores when exercise increases energy demand or contributes to negative energy balance (Rosenbaum & Leibel, 2010; Sumithran et al., 2011). This interpretation provides a physiologically coherent explanation for the pooled findings, but it should not be overextended. Hormone levels alone cannot determine whether hypothalamic appetite-regulating pathways were improved, suppressed, or compensated (Morton et al., 2006), and the included trials varied in participant characteristics, metabolic conditions, sex composition, control conditions, and exercise protocols. The clinical implication is therefore not that these findings define a specific exercise prescription, but that exercise-based weight-management programs for middle-aged and older adults with overweight or obesity may need to consider concurrent monitoring of appetite, dietary intake, and body-composition changes. Without such monitoring, compensatory energy-balance signals may partly offset the expected effects of exercise on body weight or adiposity. Accordingly, the present findings should be viewed as physiologically informative, while also highlighting the need for future trials that integrate peripheral hormone measurements with appetite ratings, objective dietary intake, body-composition changes, and, where feasible, markers of central appetite-related signaling.

### Interpretation of subgroup findings

The subgroup findings should be interpreted as exploratory evidence rather than as a basis for defining an optimal exercise prescription. For leptin, the overall pattern was relatively consistent across most exercise-related characteristics. Exercise modality did not significantly modify the leptin response (*P* for subgroup difference = 0.94), although aerobic exercise (SMD = −0.33, 95% CI [−0.53 to −0.14]) and resistance training (SMD = −0.28, 95% CI [−0.55 to −0.01]) were each associated with significant reductions. The estimate for combined exercise was in the same direction but did not reach statistical significance, which may reflect imprecision and the limited number of available comparisons rather than evidence of no effect. Similarly, no significant subgroup differences were observed by intervention duration (*P* for subgroup difference = 0.30) or weekly frequency (*P* for subgroup difference = 0.77). These findings suggest that leptin may be more closely related to the overall metabolic consequences of exercise, such as changes in adiposity, energy balance, and insulin sensitivity, than to a single isolated feature of the exercise prescription (Pasman, Westerterp-Plantenga & Saris, 1998; Kraemer, Chu & Castracane, 2002).

Session duration was the only leptin subgroup factor showing a borderline subgroup difference (*P* for subgroup difference = 0.05). Interventions lasting ≥60 min per session were associated with a larger leptin reduction (SMD = −0.49, 95% CI [−0.72 to −0.25]), whereas interventions lasting <60 min per session did not show a statistically significant effect (SMD = −0.16, 95% CI [−0.38 to 0.06]). This pattern may indicate a possible duration-related signal, as longer individual sessions could produce greater acute energy expenditure and a stronger stimulus related to fat mobilization or energy availability. However, this should not be interpreted as evidence for a definitive 60-min threshold, because exercise effects on adiposity and weight management are generally influenced by total exercise volume, intensity, adherence, and accompanying dietary factors (Donnelly et al., 2009). Session duration may be confounded by exercise intensity, total weekly volume, adherence, dietary control, baseline obesity severity, and control-condition differences. In addition, studies with unreported session duration were excluded from this subgroup analysis. Therefore, the session-duration finding should be viewed as a potentially informative but still exploratory signal.

By contrast, the subgroup pattern for ghrelin was less uniform and appeared to be shaped by a limited number of comparisons. Exercise modality did not show a statistically significant subgroup difference (*P* for subgroup difference = 0.12). Although combined exercise was associated with a significant increase in ghrelin, this estimate was based on only two comparisons and should not be interpreted as evidence that combined exercise is superior to aerobic or resistance exercise. Intervention duration also did not significantly modify the ghrelin response (*P* for subgroup difference = 0.23), and neither the ≤12-week nor the >12-week subgroup showed a statistically clear effect. In contrast, statistically significant subgroup differences were observed by weekly frequency and session duration (both *P* for subgroup difference = 0.01). The ≤3 sessions/week subgroup and the ≥60 min/session subgroup produced identical estimates (k = 2; SMD = 1.25, 95% CI [0.52–1.97]; *I^2^* = 36%), indicating substantial methodological overlap between these subgroup categories.

This overlap is central to interpreting the ghrelin subgroup findings. The ghrelin finding should not be read as two independent conclusions that lower weekly frequency and longer session duration separately increase ghrelin. Rather, the available data suggest that the signal was driven by a small set of comparisons characterized by a low-frequency and long-session intervention pattern. Physiologically, this pattern may be consistent with the possibility that longer individual exercise sessions generate a stronger short-term energy demand, which could stimulate compensatory orexigenic signaling (Schubert et al., 2014). However, because this interpretation is based on only two comparisons and because ghrelin responses are sensitive to exercise modality, energy intake, fasting status, blood-sampling time, body-composition change, and hormone measurement methods, the finding remains hypothesis-generating (Broom et al., 2009; Almesbehi et al., 2023). Overall, the subgroup analyses provide useful clues about possible modifiers of leptin and ghrelin responses, but they do not provide sufficient evidence to define a preferred exercise modality, frequency, session duration, or intervention length.

## Strengths and limitations

An important strength of this review is its focus on middle-aged and older adults with overweight or obesity, a population in whom excess adiposity, age-related body-composition changes, and metabolic dysfunction may jointly influence appetite-related hormone responses. Only randomized controlled trials were included, which strengthened the internal validity of the evidence. In addition, the use of change-based effect estimates and multiple methodological checks, including sensitivity analyses, subgroup analyses, risk-of-bias assessment, and publication-bias assessment when appropriate, helped assess the robustness and limitations of the pooled findings.

Several limitations should also be acknowledged. The included trials differed in participant profiles, metabolic conditions, sex composition, exercise prescriptions, dietary control, control conditions, and hormone measurement protocols, which may have contributed to clinical and methodological heterogeneity. In addition, participant and personnel blinding was not feasible in exercise interventions, and allocation concealment or outcome-assessment procedures were incompletely reported in several trials, which should be considered when interpreting the pooled estimates. Although subgroup analyses were conducted to explore potential modifiers, the subgroup findings for leptin should still be interpreted cautiously because participant characteristics, dietary control, and exercise prescriptions varied across trials. For ghrelin, the smaller number of comparisons and greater heterogeneity further limited our ability to interpret the independent contribution of individual exercise-prescription components, such as weekly frequency and session duration. Therefore, the subgroup findings, particularly those for ghrelin, should be regarded as exploratory rather than definitive prescription-level evidence. Publication-bias assessment also required cautious interpretation. For leptin, Egger’s test suggested potential asymmetry, but the trim-and-fill analysis did not impute any missing comparison and the pooled estimate remained unchanged. For ghrelin, the uneven funnel-plot pattern was treated as a descriptive visual finding only and was not regarded as firm evidence for or against publication bias. Accordingly, the findings support a cautious conclusion that exercise interventions are associated with changes in leptin and ghrelin concentrations in this population, while the clinical and behavioral implications of these endocrine responses require further confirmation in more standardized trials.

## Conclusions

Exercise interventions were associated with significantly lower leptin levels and higher ghrelin levels in middle-aged and older adults with overweight or obesity. The leptin finding appeared relatively consistent, whereas the ghrelin finding should be interpreted more cautiously because of the smaller number of comparisons and substantial heterogeneity. Subgroup analyses suggested that exercise-related characteristics may partly contribute to variability in leptin and ghrelin responses, but these findings remain exploratory and should not be used to define an optimal exercise prescription. Taken together, these findings indicate that exercise may be associated with changes in leptin and ghrelin concentrations in this population, although the clinical and behavioral relevance of these changes remains uncertain. Future randomized controlled trials with well-described and adequately standardized exercise protocols, clearer dietary control, standardized hormone sampling and assay procedures, and concurrent assessment of body composition, appetite, and energy intake are needed to clarify these issues.

## Supplemental Information

10.7717/peerj.21870/supp-1 Supplemental Information 1 PRISMA Checklist.

10.7717/peerj.21870/supp-2 Supplemental Information 2 Raw Data.

10.7717/peerj.21870/supp-3 Supplemental Information 3 Search strategy and supplemental figures.

## Additional Information and Declarations

### Data Availability

The following information was supplied regarding data availability:

This is a Systematic review/Meta-analysis.
