# Supplementary material for: Effects of exercise interventions on leptin and ghrelin levels in middle-aged and older adults with overweight or obesity: a systematic review and meta-analysis
Source: PeerJ. 2026 Nov 3;14:e21870. doi: 10.7717/peerj.21870 (PMC13741978; doi:10.7717/peerj.21870)
Supplement: Supplementary file 2 [file peerj-14-21870-s003.docx]

**1. Search strategy for PubMed**

#1 Obesity/overweight

("Obesity"[Mesh] OR obes*[Title/Abstract] OR "Overweight"[Mesh] OR overweight[Title/Abstract] OR "Body Mass Index"[Mesh] OR "body mass index"[Title/Abstract] OR BMI[Title/Abstract] OR "Index, Body Mass"[Title/Abstract] OR "Quetelet's Index"[Title/Abstract] OR "Quetelets Index"[Title/Abstract] OR "Quetelet Index"[Title/Abstract] OR "Index, Quetelet"[Title/Abstract] OR adiposity[Title/Abstract] OR "central obesity"[Title/Abstract] OR "abdominal obesity"[Title/Abstract])

#2 Exercise intervention

("Exercise"[Mesh] OR Exercise[Title/Abstract] OR "physical exercise"[Title/Abstract] OR "exercise training"[Title/Abstract] OR "physical activity"[Title/Abstract] OR "aerobic exercise"[Title/Abstract] OR "aerobic training"[Title/Abstract] OR "resistance exercise"[Title/Abstract] OR "resistance training"[Title/Abstract] OR "strength training"[Title/Abstract] OR "combined training"[Title/Abstract] OR "concurrent training"[Title/Abstract] OR "high-intensity interval training"[Title/Abstract] OR "high intensity interval training"[Title/Abstract] OR HIIT[Title/Abstract] OR "interval training"[Title/Abstract] OR walking[Title/Abstract] OR "brisk walking"[Title/Abstract] OR "Tai Chi"[Title/Abstract] OR "Tai Ji"[Title/Abstract] OR Qigong[Title/Abstract] OR Yoga[Title/Abstract])

#3 Leptin/ghrelin

("Ghrelin"[Mesh] OR ghrelin[Title/Abstract] OR "Appetite-Regulating Hormone"[Title/Abstract] OR "Appetite Regulating Hormone"[Title/Abstract] OR "Gastric MLTRP"[Title/Abstract] OR "Motilin-Related Peptide"[Title/Abstract] OR "Motilin Related Peptide"[Title/Abstract] OR "GHRL Protein"[Title/Abstract] OR PpMTLRP[Title/Abstract] OR "Ghrelin Precursor"[Title/Abstract] OR "Precursor, Ghrelin"[Title/Abstract] OR "Ghrelin-Obestatin Preprohormone"[Title/Abstract] OR "Ghrelin Obestatin Preprohormone"[Title/Abstract] OR "Motilin-Related Peptide Precursor"[Title/Abstract] OR "Motilin Related Peptide Precursor"[Title/Abstract] OR "Peptide Precursor, Motilin-Related"[Title/Abstract] OR "Precursor, Motilin-Related Peptide"[Title/Abstract] OR Ppghrelin[Title/Abstract] OR "Leptin"[Mesh] OR leptin[Title/Abstract] OR "Ob Gene Product"[Title/Abstract] OR "Gene Product, Ob"[Title/Abstract] OR "Ob Protein"[Title/Abstract] OR "Obese Gene Product"[Title/Abstract] OR "Gene Product, Obese"[Title/Abstract] OR "Obese Protein"[Title/Abstract])

#4 Middle-aged and older adults

("Middle Aged"[Mesh] OR "middle-aged"[Title/Abstract] OR "middle aged"[Title/Abstract] OR "Aged"[Mesh] OR "older adult"[Title/Abstract] OR "older adults"[Title/Abstract] OR elderly[Title/Abstract] OR "Postmenopause"[Mesh] OR postmenopausal[Title/Abstract] OR "post-menopausal"[Title/Abstract] OR postmenopause[Title/Abstract])

#5 Randomized controlled trials

("Randomized Controlled Trial"[Publication Type] OR random*[Title/Abstract] OR RCT[Title/Abstract])

Final search

#1 AND #2 AND #3 AND #4 AND #5

**2. Search strategy for Web of Science**

#1 Obesity/overweight

TS=(obes* OR overweight OR "body mass index" OR BMI OR "Index, Body Mass" OR "Quetelet's Index" OR "Quetelets Index" OR "Quetelet Index" OR "Index, Quetelet" OR adiposity OR "central obesity" OR "abdominal obesity")

#2 Exercise intervention

TS=(exercise OR "physical exercise" OR "exercise training" OR "physical activity" OR "aerobic exercise" OR "aerobic training" OR "resistance exercise" OR "resistance training" OR "strength training" OR "combined training" OR "concurrent training" OR "high-intensity interval training" OR "high intensity interval training" OR HIIT OR "interval training" OR walking OR "brisk walking" OR "Tai Chi" OR "Tai Ji" OR Qigong OR Yoga)

#3 Leptin/ghrelin

TS=(ghrelin OR "Appetite-Regulating Hormone" OR "Appetite Regulating Hormone" OR "Gastric MLTRP" OR "Motilin-Related Peptide" OR "Motilin Related Peptide" OR "GHRL Protein" OR PpMTLRP OR "Ghrelin Precursor" OR "Precursor, Ghrelin" OR "Ghrelin-Obestatin Preprohormone" OR "Ghrelin Obestatin Preprohormone" OR "Motilin-Related Peptide Precursor" OR "Motilin Related Peptide Precursor" OR "Peptide Precursor, Motilin-Related" OR "Precursor, Motilin-Related Peptide" OR Ppghrelin OR leptin OR "Ob Gene Product" OR "Gene Product, Ob" OR "Ob Protein" OR "Obese Gene Product" OR "Gene Product, Obese" OR "Obese Protein")

#4 Middle-aged and older adults

TS=("middle-aged" OR "middle aged" OR "older adult" OR "older adults" OR elderly OR postmenopausal OR "post-menopausal" OR postmenopause)

#5 Randomized controlled trials

TS=(random* OR RCT)

Final search

#1 AND #2 AND #3 AND #4 AND #5

**3. Search strategy for Embase**

#1
'obesity'/exp

#2
'obes*':ab,kw,ti

#3
'excess body weight'/exp

#4
'overweight':ab,kw,ti

#5
'body mass'/exp

#6
'body mass index':ab,kw,ti OR 'bmi':ab,kw,ti OR 'quetelet index':ab,kw,ti OR 'quetelet*':ab,kw,ti OR 'adiposity':ab,kw,ti OR 'central obesity':ab,kw,ti OR 'abdominal obesity':ab,kw,ti

#7
#1 OR #2

#8
#3 OR #4

#9
#5 OR #6

#10
#7 OR #8 OR #9

#11
'exercise'/exp

#12
'exercise':ab,kw,ti OR 'physical exercise':ab,kw,ti OR 'exercise training':ab,kw,ti OR 'physical activity':ab,kw,ti OR 'aerobic exercise':ab,kw,ti OR 'aerobic training':ab,kw,ti OR 'resistance exercise':ab,kw,ti OR 'resistance training':ab,kw,ti OR 'strength training':ab,kw,ti OR 'combined training':ab,kw,ti OR 'concurrent training':ab,kw,ti OR 'high-intensity interval training':ab,kw,ti OR 'high intensity interval training':ab,kw,ti OR 'hiit':ab,kw,ti OR 'interval training':ab,kw,ti OR 'walking':ab,kw,ti OR 'brisk walking':ab,kw,ti OR 'tai chi':ab,kw,ti OR 'tai ji':ab,kw,ti OR 'qigong':ab,kw,ti OR 'yoga':ab,kw,ti

#13
#11 OR #12

#14
'ghrelin'/exp

#15
'ghrelin':ab,kw,ti OR 'appetite-regulating hormone':ab,kw,ti OR 'appetite regulating hormone':ab,kw,ti OR 'gastric mltrp':ab,kw,ti OR 'motilin-related peptide':ab,kw,ti OR 'motilin related peptide':ab,kw,ti OR 'ghrl protein':ab,kw,ti OR 'ppmtlrp':ab,kw,ti OR 'ghrelin precursor':ab,kw,ti OR 'ghrelin-obestatin preprohormone':ab,kw,ti OR 'ghrelin obestatin preprohormone':ab,kw,ti OR 'motilin-related peptide precursor':ab,kw,ti OR 'motilin related peptide precursor':ab,kw,ti OR 'ppghrelin':ab,kw,ti

#16
#14 OR #15

#17
'leptin'/exp

#18
'leptin':ab,kw,ti OR 'ob gene product':ab,kw,ti OR 'ob protein':ab,kw,ti OR 'obese gene product':ab,kw,ti OR 'obese protein':ab,kw,ti

#19
#17 OR #18

#20
#16 OR #19

#21
'middle aged'/exp

#22
'middle-aged':ab,kw,ti OR 'middle aged':ab,kw,ti

#23
'aged'/exp

#24
'older adult':ab,kw,ti OR 'older adults':ab,kw,ti OR 'elderly':ab,kw,ti

#25
'postmenopause'/exp

#26
'postmenopausal':ab,kw,ti OR 'post-menopausal':ab,kw,ti OR 'postmenopause':ab,kw,ti

#27
#21 OR #22

#28
#23 OR #24

#29
#25 OR #26

#30
#27 OR #28 OR #29

#31
'randomized controlled trial'/exp

#32
'random*':ab,kw,ti OR 'rct':ab,kw,ti

#33
#31 OR #32

#34
#10 AND #13 AND #20 AND #30 AND #33

**4. Search strategy for Cochrane Library**

#1 Obesity/overweight

MeSH descriptor: [Obesity] explode all trees

#2

obes*:ti,ab,kw

#3

MeSH descriptor: [Overweight] explode all trees

#4

overweight:ti,ab,kw

#5

MeSH descriptor: [Body Mass Index] explode all trees

#6

("body mass index" OR BMI OR "Index, Body Mass" OR "Quetelet's Index" OR "Quetelets Index" OR "Quetelet Index" OR "Index, Quetelet" OR adiposity OR "central obesity" OR "abdominal obesity"):ti,ab,kw

#7

#1 OR #2

#8

#3 OR #4

#9

#5 OR #6

#10

#7 OR #8 OR #9

#11 Exercise intervention

MeSH descriptor: [Exercise] explode all trees

#12

(exercise OR "physical exercise" OR "exercise training" OR "physical activity" OR "aerobic exercise" OR "aerobic training" OR "resistance exercise" OR "resistance training" OR "strength training" OR "combined training" OR "concurrent training" OR "high-intensity interval training" OR "high intensity interval training" OR HIIT OR "interval training" OR walking OR "brisk walking" OR "Tai Chi" OR "Tai Ji" OR Qigong OR Yoga):ti,ab,kw

#13

#11 OR #12

#14 Leptin/ghrelin

MeSH descriptor: [Ghrelin] explode all trees

#15

ghrelin:ti,ab,kw

#16

MeSH descriptor: [Leptin] explode all trees

#17

leptin:ti,ab,kw

#18

#14 OR #15

#19

#16 OR #17

#20

#18 OR #19

#21 Middle-aged and older adults

MeSH descriptor: [Middle Aged] explode all trees

#22

("middle-aged" OR "middle aged"):ti,ab,kw

#23

MeSH descriptor: [Aged] explode all trees

#24

("older adult" OR "older adults" OR elderly):ti,ab,kw

#25

MeSH descriptor: [Postmenopause] explode all trees

#26

(postmenopausal OR "post-menopausal" OR postmenopause):ti,ab,kw

#27

#21 OR #22

#28

#23 OR #24

#29

#25 OR #26

#30

#27 OR #28 OR #29

#31 Randomized controlled trials

("Randomized Controlled Trial" OR random* OR RCT):ti,ab,kw

Final search

#10 AND #13 AND #20 AND #30 AND #31

**5. Search strategy for CNKI**

#1 Obesity/overweight

超重 OR 肥胖 OR 体重指数

#2 Exercise intervention

运动 OR 锻炼 OR 有氧运动 OR 抗阻运动 OR 联合运动 OR 太极拳 OR 健身气功 OR 八段锦

#3 Randomized controlled trials

随机对照研究 OR 随机对照实验

Final search

#1 AND #2 AND #3

**Supplementary Figure S1. Sensitivity analysis for leptin.**

**Supplementary Figure S2. Sensitivity analysis for ghrelin.**
